# Data on characterization of metalloporphyrin-mediated HO-1 and DAF induction in rat glomeruli and podocytes

**DOI:** 10.1016/j.dib.2018.11.108

**Published:** 2018-11-26

**Authors:** Pu Duann, Pei-Hui Lin, Elias A. Lianos

**Affiliations:** a Veterans Affairs Medical Center, Salem VA 24153, USA; b Davis Heart and Lung Research Institute, and Department of Surgery, Division of Cardiac Surgery, The Ohio State University Wexner Medical Center, Columbus, OH 43210, USA; c Veterans Affairs Medical Center, Salem VA and Virginia Tech. Carilion School of Medicine, Roanoke, VA 24153, USA

**Keywords:** Glomerulus, HO-1, DAF, Podocyte, Metalloporphyrins

## Abstract

The data presented pertain to a research article titled "Heme Oxygenase 1 Up-Regulates Glomerular Decay Accelerating Factor Expression and Minimizes Complement Deposition and Injury" (Detsika et al., 2016). The present work provides additional data on induction and immunolocalization of heme oxygenase (HO)-1 (an antioxidant enzyme) and decay-accelerating factor (DAF) (a complement activation inhibitor) in isolated rat glomeruli and in glomerular epithelial cells (podocytes) in response to Iron Protoporphyrin IX (FePP, heme), and to non-iron protoporphyrins (PPs) with varying metal functionalities (ZnPP, SnPP), including a metal-devoid PP. Induction and immuno-localization of HO-1 and DAF in response to these metalloporphyrins (MP) were assessed using western blot analyses and confocal microscopy in isolated glomeruli and in cultured podocytes. These analyses identified podocytes as a major localization site of HO-1 and DAF induction in response to the aforementioned MPs. Effects of these MPs on a key glomerular structural protein, Nephrin, are also reported. The data identify MPs most and least capable of inducing DAF and reducing Nephrin expression and provide clues into expected outcomes of animal studies assessing MP efficacy in upregulating the cytoprotective proteins HO-1 and DAF.

**Specifications table**

**Table:** 

Subject area	Cell physiology
More specific subject area	Renal glomeruli and podocyte cell culture, HO-1 and DAF induction upon metalloporphyrin (MP) treatment, glomerular structural integrity
Type of data	Images
How data was acquired	Confocal laser scanning microscopy and Immuno-blotting
Data format	Raw and analyzed data
Experimental factors	Isolated renal glomeruli or podocyte cells cultures stimulated with MP.
Experimental features	Glomeruli were isolated from rat kidney by differential sieving and incubated with various MPs to induce HO-1 and DAF expression. Expression and subcellular localization were analyzed with immunostaining, confocal laser scanning microscopy, and western blotting. Cultured glomerular podocytes were incubated with iron protoporphyrin (FePP, heme), and cellular HO-1 distribution was analyzed with differential subcellular fractionation, western blotting, immunostaining and confocal laser scanning microscopy analysis.
Data source location	Davis Heart and Lung Research Institute, The Ohio State University Wexner Medical Center, Columbus, OH 43210, USA
Data accessibility	The data are available with this article.
Related research article	*Detsika MG, et al*. Heme Oxygenase 1 Up-Regulates Glomerular Decay Accelerating Factor Expression and Minimizes Complement Deposition and Injury. Am J Pathol. 2016 Nov;186(11):2833–2845 [1].

**Value of the data**

• The data demonstrate that in glomeruli incubated with MPs, HO-1 and DAF induction immunolocalizes in podocytes, which can be of value to investigators interested in identifying protective mechanisms of these terminally differentiated non-replicative cells under conditions of injury.
• HO-1 translocation to the nucleus in cultured podocytes incubated with FePP (heme) is a novel finding as this translocation could regulate transcription factors of cytoprotective genes such as DAF. The data also demonstrate that, depending on their metal moiety, MPs can reduce expression of glomerular nephrin, which is a key structural protein of the glomerular capillary barrier (slit diaphragm) to protein. This adverse effect can be of value to investigators interested in design of MP-based therapeutic strategies in kidney disease.

## 1 Data

For subcellular localization and expression analyses of HO-1 or DAF, isolated rat glomerulus was induced with FePP and immuno-stained and analyzed with confocal microscopy. The relative levels of HO-1 and DAF induction by FePP on isolated glomeruli or cultured podocytes were further analyzed by western blot. For changes of HO-1 in subcellular distribution upon FePP stimulation was analyzed by confocal microscopy and western blot. The levels of DAF and nephrin expression in response to additional MP on isolated glomerulus were analyzed with western blot.

**Fig. 1:**
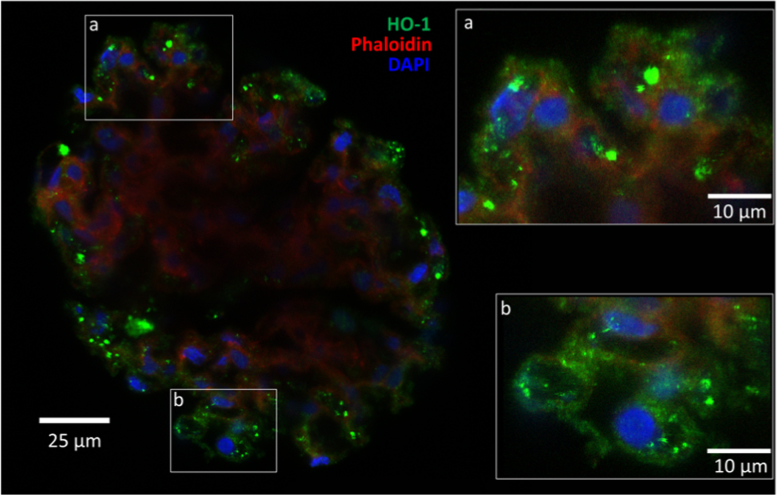
Immunolocalization of FePP-mediated HO-1 induction in glomeruli. Distribution of HO-1 induction assessed by immunolabeling and Confocal laser scanning microscopy (CLSM). Isolated glomeruli preparation were treated with the natural HO substrate/inducer Iron Protoporphyrin IX [FePP (heme): 5 µM, 6 h incubation]. HO-1 induction was detected in cytoplasm of cells at the periphery of the glomerulus (podocytes) which is apparent and best seen in inserts a and b.

**Fig. 2:**
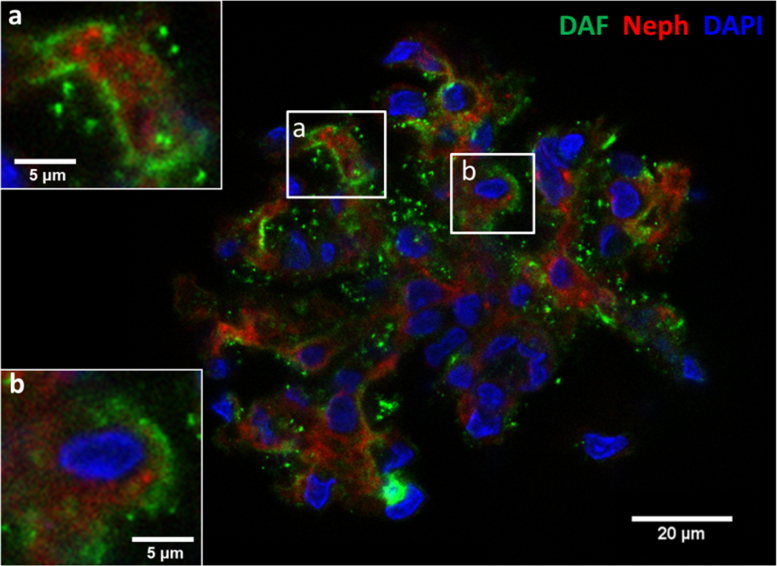
Imunolocalization of FePP-mediated DAF induction in glomeruli. Distribution of DAF induction assessed by immunolabeling and CLSM in single glomerulus in response to FePP (5 µM, 6 h incubation) is shown. DAF induction concentrated in membranes of cells at the periphery of the glomerulus (podocytes), best seen at high magnification in inserts a and b.

**Fig. 3:**
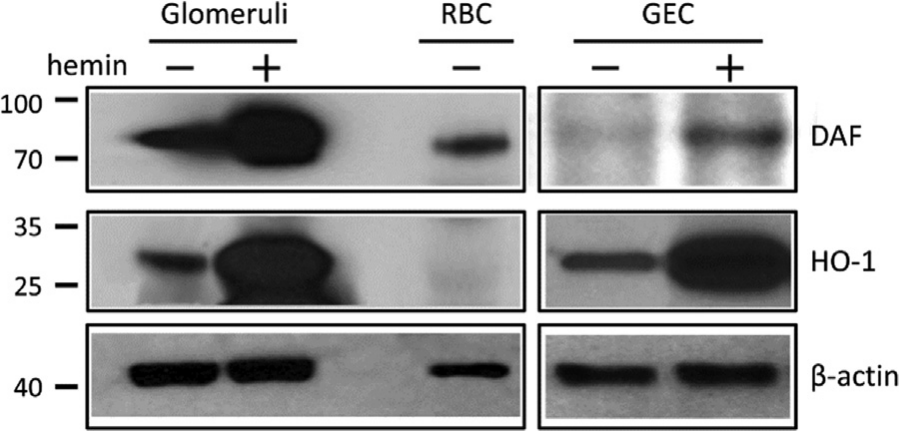
Verification of HO-1 and DAF induction in isolated glomeruli and podocytes incubated with FePP. Magnitude of HO-1 and DAF induction in response to FePP (5 µM, 6 h incubation) assessed by western blot analysis. HO-1 and DAF protein levels increased in both glomeruli and cultured glomerular epithelial cells (podocytes). There was comparable HO-1 induction in glomeruli and podocytes. However, DAF induction was less robust in podocytes. RBC: red blood cell protein extract used as a positive control (a rich source of membrane bound DAF).

**Fig. 4:**
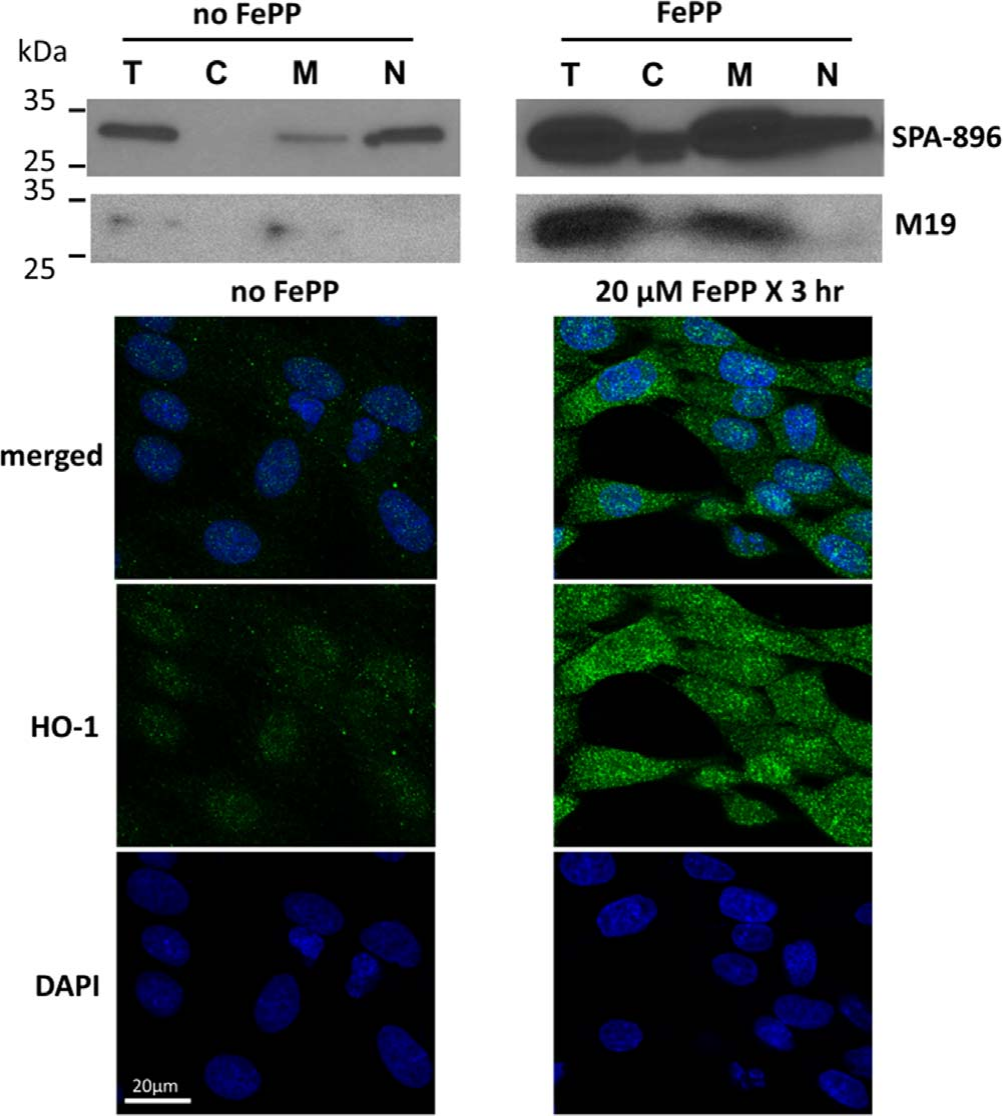
HO-1 induction in podocytes involves nuclear translocation. Subcellular distribution of HO-1 induction in cell fractions of cultured podocytes exposed to FePP (25 µM, 3 h incubation) is shown. FePP significantly increased HO-1 protein in all cell fractions including total cell lysates (T), cytoplasmic (C), membrane (M) and nuclear (N) fractions (Fig. 4, upper panel). The most pronounced induction localized in total membrane (M) fraction where HO-1 is known to be anchored to the smooth endoplasmic reticulum membrane (sER) via its C terminus, that resides within the sER lumen. HO-1 translocation to the nucleus requires proteolytic cleavage and releases the HO-1 N-terminus into the cytosol. We used a N-terminus antibody (Ab) against a 30-residue synthetic peptide lacking the membrane spanning region (clone SPA-896) obtained from. Enzo Life Sciences, and an antibody specific for the C-terminus amino acid sequence of the HO-1 protein (clone M19) obtained from Santa Cruz Biotechnology. Use of these antibodies demonstrated that HO-1 immunoreactivity detected in nuclear fractions (SPA-896 Ab) following FePP treatment (Fig. 4, upper panel) could not be accounted for by presence of the C terminus (M19 Ab) and suggests nuclear translocation of the N terminus, which is generated by proteolytic cleavage in cytosol. Further validation of localization of the N terminus peptide in the nucleus was obtained by immunostaining (SPA-896 Ab) and confocal microscopy as shown in Fig. 4 (lower panel, top right-merged image).

**Fig. 5:**
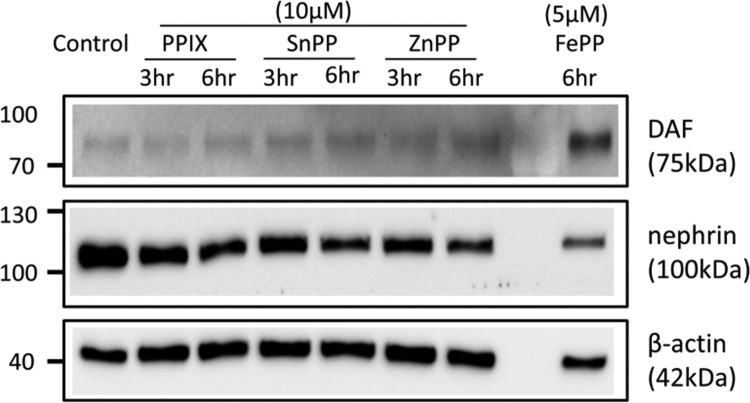
Effect of Metalloporphyrins on glomerular DAF and nephrin expression. Isolated glomeruli were incubated with 10 µM of either ZnPP, SnPP or PPIX. FePP (5 µM) incubations were used as a positive control. FePP was the most potent MP in inducing glomerular DAF (5 µM, 6 h incubations). SnPP and ZnPP at 10 µM (3 or 6 h incubations) also induced DAF while the metal-devoid, PPIX, had no significant effect. All MPs reduced nephrin expression, the most potent being FePP while the least potent was the metal-devoid PPIX.

**Fig. 6:**
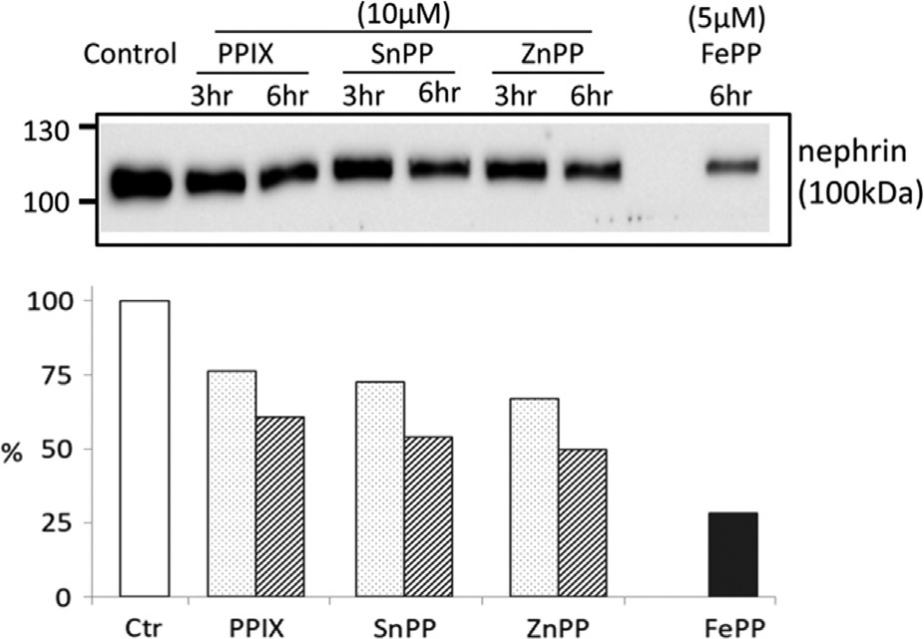
MPs reduce glomerular Nephrin expression. Isolated glomeruli were incubated with ZnPP, SnPP or PPIX. FePP (5 µM, 6 h incubations) significantly reduced glomerular Nephrin levels. SnPP, ZnPP and PPIX (10 µM, 3 or 6 h incubations) also reduced Nephrin expression (most apparent at 6 h incubations) but to a lesser extent compared to FePP. The relative potency of MPs on glomerular Nephrin expression (expressed as percent of control value) is shown in the bar graph.

The data presented demonstrate that: (i) In the whole glomerulus, FePP (heme, hemin) induces HO-1 and DAF and this induction localizes in podocytes as shown by confocal microscopy (Fig. 1, Fig. 2). (ii) The magnitude of HO-1 induction (assessed by western blot analysis) in response to FePP is comparable in isolated glomeruli and in cultured podocytes (Fig. 3), in which FePP also caused HO-1 translocation to the nucleus (Fig. 4). In contrast, DAF induction was far less robust in cultured podocytes compared to that in whole glomeruli (Fig. 3), and (iii) The potency of MPs in inducing glomerular DAF depends of metal functional moiety with FePP being the most potent (Fig. 5). However, this MP was also the most potent in reducing glomerular Nephrin expression (Fig. 6), which is an undesirable effect.

## 2 Experimental design, materials and methods

Isolated rat glomerulus or cultured podocytes were stimulated with various MPs, including FePP, PPIX, SnPP and ZnPP, and the expression and subcellular localization of DAF or HO-1 were analyzed with confocal microscopy with anti-DAF or with differential anti-HO-1 antibodies, subcellular fraction and western blots.

### 2.1 Reagents

Anti–Hmox-1 (HO-1) polyclonal antibodies were used for experiments. Anti-C terminus of human HO-1 antibody (M-19) was from Santa Cruz Biotechnology (Dallas, TX). Anti-N terminus of human HO-1 antibody (SPA-896) was from Enzo Life Sciences (New York, NY). Anti-rat DAF antibody (clone RDIII-7) was purchased from Hycult (Plymouth Meeting, PA) and anti β-actin antibody from Sigma-Aldrich (St. Louis, MO). Hemin, zinc protoporphyrin, protoporphyrin IX (PPIX), and Tin protoporphyrin were purchased from Tocris Bioscience (Minneapolis, MN). Insulin and transferrin were from Sigma- Aldrich (St. Louis, MO).

### 2.2 Isolation of glomeruli

Glomeruli were isolated after whole body perfusion. This was performed by injecting (#16 needle) 250 ml of pre-chilled heparinized PBS saline (0.2% heparin salt) in the left ventricle while making a notched-cut in the liver for blood drainage. Perfusion continued until kidney color was pale. Glomeruli were isolated by differential sieving as previously described (1). Glomeruli retained atop the 75 μm sieve were retrieved and cultured on 5% Matrigel-coated glass bottomed 35 mm culture dishes (MatTek Corporation) in phenol red-free RPMI-1640 medium supplemented with 10% FBS, 100 U/ml penicillin and 0.1 mg/ml streptomycin, and incubated at 37 °C under 5% CO_2_.

### 2.3 Immunostaining and confocal microscopy

Isolated glomeruli were fixed (4% paraformaldehyde, 30 min), permeabilized with 0.2% triton X-100 for 30 min, and blocked with a blocking solution (PBS, 3% IgG-free and protease-free bovine serum albumin Fraction V, Jackson Immuno Research Laboratories, West Grove, PA) for 1 h at room temperature (RT). Fixed glomeruli were incubated over night at 4 °C with the following antibodies diluted in blocking solution: monoclonal mouse anti-rat gpi-anchored DAF (1:500, clone RDIII-7, Hycult, Plymouth Meeting, PA), polyclonal rabbit anti-human HO-1 antibody (1:300, Enzo Life Sciences, New York, NY), polyclonal rabbit anti-rat nephrin (1:300, Abcam, Cambridge, MA). Glomeruli were also stained with Phalloidin-Alexa Fluor 647 solution (1:500, Thermo Fisher Scientific, Waltham, MA) for F-actin staining (shown in Fig. 1). After washing (3 times × 15 min with PBS), isolated glomeruli were incubated with Alexa Fluor-labelled secondary antibodies (1:300, Thermo Fisher Scientific, Waltham, MA) for 1 h at RT. Nuclear staining was done using DAPI (4׳,6-Diamidino-2-Phenylindole, Dihydrochloride, Sigma, St. Louis, MO).

Immunostained glomeruli were placed in glass-bottom dish (MatTek Corporation, Ashland, MA) in phosphate buffered saline (PBS) to reduce the background fluorescent signal and imaged using a Confocal Laser Scanning Microscope (CLSM, Zeiss LSM 780) using a 63× oil-immersion objective (numerical aperture 0.9, 1 Airy unit). A z-stack with two channels, one for DAF (Alexa Fluor 488 - excitation 495 nm, emission 519 nm) and one for Nephrin (Alexa Fluor 546 - excitation 556 nm, emission 573 nm), was collected for each of the isolated glomeruli examined (*n* = 80, for a total of three experimental repeats). We also collected a single image of stained nuclei (DAPI: excitation 405 nm, emission 413 nm to 467 nm) to confirm that objects examined were cells (objects without a nucleus were discarded). Based on the resolution for the 63× objective (*X* = 134.7 µm, *Y* = 134.7 µm and *Z* = 70 µm for 488 nm wavelength), we defined our acquisition fluorescence voxel dimensions in *X*, *Y* and *Z* at 207.57 nm (*X*) × 207.57 μm (*Y*) × 68.34 μm (*Z*) (1024 pixels × 1024 pixels in *X* and *Y*, up to 40 frames in *Z*). Each z-frame in the z-stacks was exported as a 4.8 MB TIFF image with resolution of 1024 pixels × 1024 pixels (212.55 μm × 212.55 μm) and 16 bits per pixel.

### 2.4 Podocyte cultures

Podocytes were cultured from isolated glomeruli of Sprague-Dawley rats (Harlan, Indianapolis, IN) as previously described and were a kind gift of Prof. BS Kasinath (Division of Nephrology, University of Texas, San Antonio) [2]. Cells were maintained in culture with media containing 50% DMEM, 50% Ham׳s F-12 (Gibco BRL, Grand Island, NY), 5% fetal calf-serum, 5 g/ml insulin, 5 g/ml transferrin, and 5 g/ml selenium at 37 °C in 5% CO_2_. Podocytes at passages 8 to 12 were used for experiments.

### 2.5 Protein extraction of samples

Isolated glomeruli or cultured podocytes were washed twice with PBS and then re-suspended in RIPA lysis buffer (Thermo Fisher Scientific) plus protease inhibitor cocktail (Sigma) for 10 min. Glomerular protein extraction were done by three-cycle of freeze-thawing on dry ice and extracted with pestle homogenizer with disposable polypropylene pestles (Thermo Fisher Scientific). Lysates were centrifuged (20,000 × *g*, 20 min. at 4 °C) and protein containing supernatant was collected. Protein concentration was determined using a Pierce Bradford Assay kit (Thermo Fisher Scientific).

### 2.6 Cell fractionation

Cultured podocytes were collected and washed twice with PBS. Total protein lysates were prepared with RIPA lysis buffer as above. For differential subcellular fractionation, cell pellet was first resuspended in hypotonic buffer A (100 mM Tris–HCl, pH 7.2, 0.5 mM MgCl_2_) with 0.1% saponin and protease inhibitor cocktails (Sigma) under rocking for 10 min and centrifuged (14,000 rpm, 5 min.) at 4 °C. The collected supernatant was the cytosol fraction. The resulting pellet was then extracted with 1% triton X-100 in buffer A containing protease inhibitor cocktails, and centrifuged as above at 4 °C. This supernatant was the membrane fraction containing plasma membrane and endoplasmic reticulum (ER) membranes. The resulting pellet was the nuclear fraction which was subsequently extracted with RIPA lysis buffer and centrifuged.

### 2.7 Western blots

Protein samples were loaded (10 μg per lane) and separated on precast gels, 10% MINI-PROTEAN TGX™ (Bio‐Rad Laboratories, Hercules, CA). Protein gel was transferred to PVDF membrane for immunoblotting. After a blocking step (5% non-fat milk powder in 0.05% Tween-20 supplemented Tris-buffered saline (TBST), for 30 min), membranes were incubated with either of the following primary antibodies: anti-human HO-1 antibody against N-terminus (clone SPA-896), anti-human HO-1 antibody against C-termus (clone M19), anti-rat DAF antibody (clone RDIII-7), rabbit anti-Nephrin polyclonal antibody. HRP-coupled secondary antibodies were goat anti‐mouse (1: 5000), goat anti-rabbit (1:5000) or rabbit anti-goat (1:5000). The Pierce ECL Western Blotting Substrate (Thermo Fisher Scientific) were used for visualization.
